# Distribution of Serotonin-Immunoreactive Neurons in the Brain and Gnathal Ganglion of Caterpillar *Helicoverpa armigera*

**DOI:** 10.3389/fnana.2019.00056

**Published:** 2019-05-28

**Authors:** Qing-Bo Tang, Wei-Wei Song, Ya-Jun Chang, Gui-Ying Xie, Wen-Bo Chen, Xin-Cheng Zhao

**Affiliations:** Department of Entomology, College of Plant Protection, Henan Agricultural University, Zhengzhou, China

**Keywords:** *Helicoverpa armigera*, serotonin, immunoreactivity, wide-field neurons, commissure, neuropils, brain, gnathal ganglion

## Abstract

Serotonin (5-hydroxytryptamine, 5-HT) is an important biogenic amine that acts as a neural circuit modulator. It is widespread in the central nervous system of insects. However, little is known about the distribution of serotonin in the nervous system of the cotton bollworm *Helicoverpa armigera*. In the present study, we performed immunohistochemical experiments with anti-serotonin serum to examine the distribution of serotonin in the central nervous system of *H. armigera* larvae. We found about 40 serotonin-immunoreactive neurons in the brain and about 20 in the gnathal ganglion. Most of these neurons are wide-field neurons giving rise to processes throughout the neuropils of the brain and the gnathal ganglion. In the central brain, serotonin-immunoreactive processes are present bilaterally in the tritocerebrum, the deutocerebrum, and major regions of the protocerebrum, including the central body (CB), lateral accessory lobes (LALs), clamps, crepine, superior protocerebrum, and lateral protocerebrum. The CB, anterior ventrolateral protocerebrum (AVLP), and posterior optic tubercle (POTU) contain extensive serotonin-immunoreactive process terminals. However, the regions of mushroom bodies, the lateral horn, and protocerebral bridges (PBs) are devoid of serotonin-immunoreactivity. In the gnathal ganglion, the serotonin-immunoreactive processes are also widespread throughout the neuropil, and some process projections extend to the tritocerebrum. Our results provide the first comprehensive description of the serotonergic neuronal network in *H. armigera* larvae, and they reveal the neural architecture and the distribution of neural substances, allowing us to explore the neural mechanisms of behaviors by using electrophysiological and pharmacological approaches on the target regions.

## Introduction

Serotonin (5-hydroxytryptamine, 5-HT) is a biogenic amine that is widely present in both invertebrate and vertebrate animal species (Vleugels et al., 2015). In insects, as in vertebrates and other invertebrates, serotonin is an important monoamine neurotransmitter that functions as a neural circuit modulator (Nässel, 1988; Qi et al., 2014; Vleugels et al., 2015). Antiserum against serotonin has been available for three decades, and the immunohistochemical experiments to investigate the distribution of serotonin in the nervous system have been performed in a large number of insect species, including locusts, mantes, cockroaches, bugs, beetles, ants, wasps, bees, flies, mosquitoes, and moths (Bishop and O’Shea, 1983; Tyrer et al., 1984; Lange et al., 1988; Nässel, 1988; Homberg and Hildebrand, 1989a, b; Breidbach, 1990; Boleli and Paulino-Simões, 1999; Leitinger et al., 1999; Settembrini and Villar, 2004; Dacks et al., 2006; Liu et al., 2011; Huser et al., 2012; van der Woude and Smid, 2017). Serotonin is distributed throughout the nervous system, including both peripheral and central regions. Physiological and behavioral experiments have demonstrated that serotonin is involved in vision (Paulk et al., 2009), olfaction (Linn and Roelofs, 1986; Gatellier et al., 2004; Dacks et al., 2008; Kloppenburg and Mercer, 2008; Ellen and Mercer, 2012; Muscedere et al., 2012), hearing (Andrés et al., 2016), feeding (Ali, 1997; Kaufmann et al., 2004; Orchard, 2006; Haselton et al., 2009; French et al., 2014; Schoofs et al., 2018), circadian behavior (Hinks, 1967; Page, 1987; Cymborowski, 1998; Chen et al., 1999; Tomioka, 1999; Saifullah and Tomioka, 2002; Yuan et al., 2005; Giese et al., 2018), aggregation (Anstey et al., 2009; Rogers and Ott, 2015), and learning and memory (Sitaraman et al., 2008, 2012; Fernández et al., 2012).

The cotton bollworm *Helicoverpa armigera* (Hübner; Lepidoptera: Noctuidae), is an important agricultural pest in the world, feeding on more than 160 plant species (Fitt, 1989; Ma et al., 2016). The damage of *H. armigera* to plants occurs during its larval stage. Feeding behavior of *H. armigera* larvae is mediated to a large extent by the gustatory sensillum, which detects palatable and unpalatable compounds in plants to acquire nutrients and avoid the toxins (Zhou et al., 2010). Behavioral and gustatory electrophysiological studies revealed that feeding experiences could induce changes in feeding preference of *H. armigera* for host plants (Zhou et al., 2010; Ma et al., 2016; Wang et al., 2017). Such feeding preference and plasticity is mediated by the signal transduction of the gustatory pathway and the modulation of the neural substance in the central nervous system (Glendinning et al., 1999, 2001). However, to date little is known about the neural substances in the nervous system of *H. armigera*.

In the present study, we performed immunohistochemistry with anti-serotonin serum to examine the distribution of serotonin in the central nervous system of *H. armigera* larvae. We provide the first comprehensive description of the serotonergic neuronal network in *H. armigera* larvae, which is the basic knowledge about the neural architecture and the distribution of neural substance and improves our understanding of the neural mechanism of behaviors, such as, host selection, navigation, and feeding preference and plasticity. In addition, the findings are essential to develop novel methods to control the pest by modulating the insect behaviors with the neural substance of serotonin.

## Materials and Methods

### Insects

Larvae of *H. armigera* were reared on an artificial diet in the laboratory under 16/8 h light/dark, at 27°C and 70% relative humidity. Two-day-old 5th instar larvae were used for the experiments. No permission from the ethics committee is required for experiments on *H. armigera* according to the laws on animal welfare in China.

### Immunocytochemistry for Serotonin and Synapsin

Brains and gnathal ganglia of *H. armigera* larvae were dissected out in fresh Ringer’s solution [150 mM NaCl, 3 mM CaCl_2_, 3 mM KCl, 25 mM Sucrose, and 10 mM N-tris (hydroxymethyl)-methyl-2-amino-ethanesulfonic acid, pH 6.9] on ice. Immunostaining with anti-serotonin was performed to examine the distribution of serotonin-immunoreactive neurons in the brain and the gnathal ganglion. To visualize the outline of the neuropil structure, immunostaining with anti-synapsin was also performed. The immunostaining of serotonin and synapsin was conducted following previously described procedures (Zhao et al., 2016). The dissected brains and gnathal ganglia were fixed in 4% paraformaldehyde for 1–2 h at room temperature, and they were rinsed four times with phosphate-buffered saline (PBS; 684 mM NaCl, 13 mM KCl, 50.7 mM Na_2_HPO_4_, 5 mM KH_2_PO_4_, pH 7.4) for 15 min. To minimize the non-specific staining, the rinsed brains and gnathal ganglia were pre-incubated in 5% normal goat serum (NGS, Sigma, St. Louis, MO, USA) in PBS (0.1 M, pH 7.4) containing 0.5% Triton X-100 (PBSX) for 3 h at room temperature. Next, the samples were incubated with anti-serotonin serum (1:5,000; Immunostar Inc., Hudson, WI, USA) and anti-SYNORF1 serum (1:200; Developmental Studies Hybridoma Bank, University of Iowa, Iowa City, IA, USA) in PBSX containing 5% NGS for 5 days at 4°C. The specificity of anti-SYNORF1 had been confirmed by both western blot and immunohistochemistry (Godenschwege et al., 2004). The labeling of the synaptic neuropil with this antibody had been reported previously in a large number of insect species, including heliothine moths (Zhao et al., 2016). The specificity of the anti-serotonin had been tested in heliothine moth by preadsorption of lyophilized serotonin creatine sulfate coupled to bovine serum albumin (Immunostar) at a concentration of 20 μg/ml (Zhao and Berg, 2009). The preadsorption abolished all immunostaining with anti-serotonin serum in the brain of heliothine moths. Following six rinse in PBS for 20 min, the brains and gnathal ganglia were incubated in secondary Cy2-conjugated goat anti-mouse antibodies (1:500; Invitrogen, Eugene, OR, USA) and Cy5-conjugated goat anti-rabbit antibodies (1:500; Invitrogen) in PBSX for 3 days at 4°C in the dark. After being rinsed in PBS 6 × 20 min, the brains and gnathal ganglia were dehydrated in an ethanol series (50%, 70%, 90%, 96%, and 2 × 100%, each 10 min), cleared in methyl salicylate, and mounted in Permount.

### Image Acquisition, Three-Dimensional Reconstruction, and Image Processing

The images of serial confocal stacks were acquired by using a confocal laser scanning microscope (LSM710, META Zeiss, Jena, Germany) with an objective of 10× (Plan-Neofluar 10×/0.3) and 20× (Plan-Neofluar 20×/0.5l). To excite Cy2 and Cy5, a 488-nm l Argon laser and a 633 nm HeNe laser were used, respectively. The resolutions of images are 1,024 × 1,024 pixels and the intervals are 2–3 μm.

To create the three-dimensional models of brains, gnathal ganglia, cell bodies, and thick fiber of serotonin-immunoreactive neurons, the confocal stacks were imported into the visualization software AMIRA (AMIRA 5.3, Visage Imaging, Fürth, Germany). The Segmentation editor tool was used to label the neuropils and the Skeleton tree was used to trace the thick neuron fibers (Zhao et al., 2017).

Necessary adjustments for brightness and contrast of the images were made in Adobe Photoshop, and the final figure panels were edited in Adobe Illustrator CS2 (Adobe System, San Jose, CA, USA). Terminology and abbreviations for neuropil structures suggested by Ito et al. (2014) were used for the brain and the gnathal ganglion of *H. armigera* larvae. The axis of neuropil is taken as the axis of the insect body. The orientation of the larval brain is about 90° different from that of adult insects, however, the neuropil structures in the larval brain are given the same names as the corresponding structures in adult insects, regardless of the orientation.

## Results

**Figure 1:**
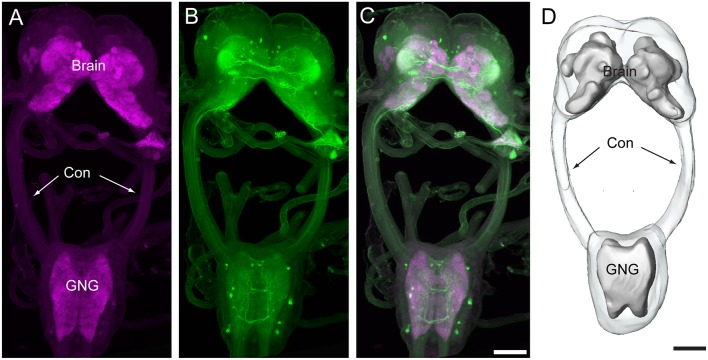
Confocal images and three-dimensional reconstruction of the brain and the gnathal ganglion of *Helicoverpa armigera* larvae. **(A)** Confocal image showing the neuropils of the brain and the gnathal ganglion. **(B)** Confocal image showing the serotonin-immunoreactive neurons in the brain and the gnathal ganglion. **(C)** Merged confocal image showing the neuropil (magenta) and the serotonin-immunoreactive neurons (green). **(D)** Three-dimensional reconstruction of the brain and the gnathal ganglion. Con, connective; GNG, gnathal ganglion. Scale bars, 100 μm.

In total, immunocytochemical experiments for synapsin and serotonin were performed on 24 preparations. Of these, 16 were successfully stained, and seven were used to obtain the confocal images and to count the number of serotonin-immunoreactive neurons in the brain and the gnathal ganglion. The brains and gnathal ganglia of *H. armigera* larvae were connected by a pair of circumesophageal connectives (Figure 1). The serotonin-immunoreactive neurons were reliably distinguished by their cell body positions in the cell body rind of the brain and the gnathal ganglion. The nomenclature of neurons used in this report is based on the cell body position.

### General Anatomy of the *H. armigera* Larval Brain

**Figure 2:**
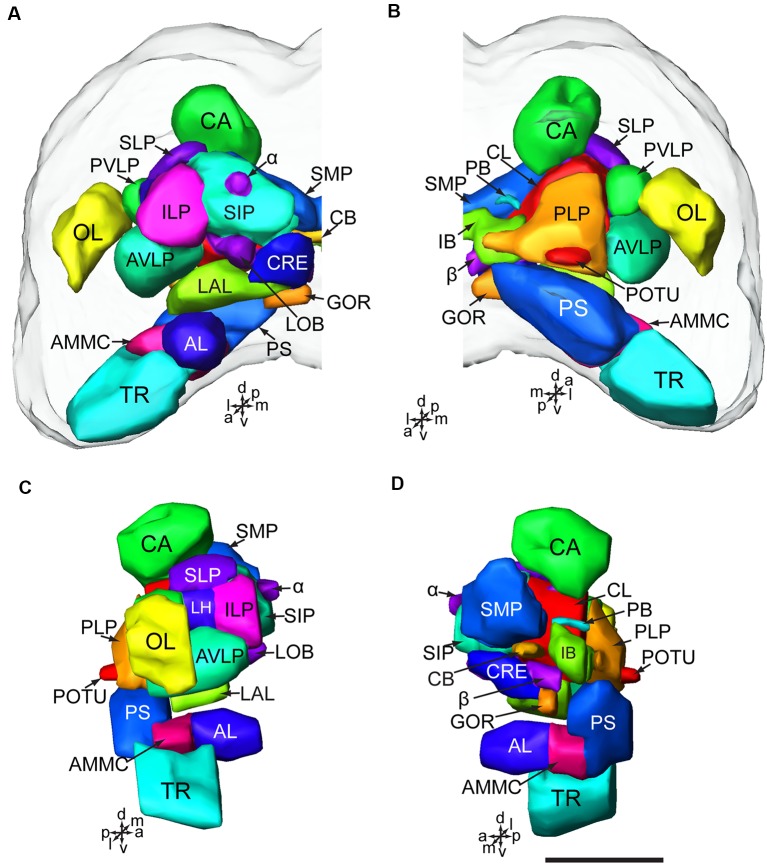
Brain composition of *H. armigera* larvae. **(A)** Three-dimensional reconstuctions of one brain hemisphere in frontal view; **(B)** in posterior view; **(C)** in lateral view; **(D)** in sagittal view. α, alpha lobe; AL, antennal lobe; AMMC, antennal mechanosensory and motor center; AVLP, anterior ventrolateral protocerebrum; *β*, belta lobe; CA, calyx; CB, central body CL, clamp; CRE, crepine; GOR, gorget; IB, inferior bridge; ILP, inferior lateral protocerebrum; LAL, lateral accessory lobe; LH, lateral horn; LOB, mushroom body lobes; OL, optic lobe; PB, protocerebral bridge; PLP, posterior lateral protocerebrum; POTU, posterior optic tubercle; PS, posterior slope; PVLP, posterior ventrolateral protocereburm; SIP, superior intermediate protocerebrum; SLP, superior lateral protocerebrum; SMP, superior medial protocerebrum; TR, tritocerebrum. Directions: a, anterior; d, dorsal; l, lateral; m, medial; p, posterior, v, ventral. Scale bar, 100 μm.

**Figure 3:**
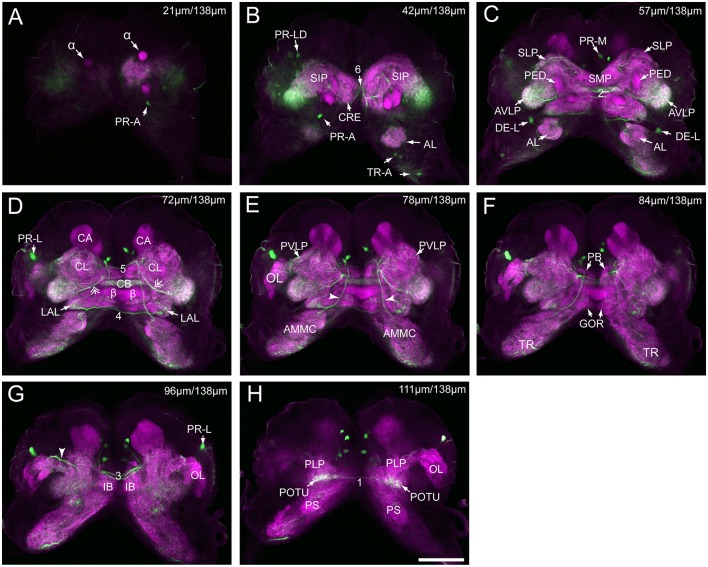
Serial confocal images from anterior to posterior showing the serotonin- immunoreactive neurons (green) and the brain neuropils (magenta) of *Helicoverpa armigera* larvae. **(A)** Confocal image of the brain section at a depth of 21 μm of total 138 μm; **(B)** at 42 μm; **(C)** at 57 μm; **(D)** at 72 μm. Double arrows indicate the processes from the cells in the cluster of PR-M extending to the AVLP; **(E)** at 78 μm. Arrowheads indicate the processes of the cell in the cluster of DE-L; **(F)** at 84 μm; **(G)** at 96 μm. Arrowhead indicates the processes of cells in the cluster of PR-L; **(H)** at 111 μm. α, alpha lobe; AL, antennal lobe; AMMC, antennal mechanosensory and motor center; AVLP, anterior ventrolateral protocerebrum; *β*, belta lobe; CA, calyx; CB, central body; CL, clamp; CRE, crepine; GOR, gorget; IB, inferior bridge; ILP, inferior lateral protocerebrum; LAL, lateral accessory lobe; LOB, mushroom body lobes; OL, optic lobe; PB, protocerebral bridge; PLP, posterior lateral protocerebrum; POTU, posterior optic tubercle; PS, posterior slope; PVLP, posterior ventrolateral protocereburm; SIP, superior intermediate protocerebrum; SLP, superior lateral protocerebrum; SMP, superior medial protocerebrum; TR, tritocerebrum. DE-L, PR-A, PR-L, PR-LD, PR-M, and TR-A are cell clusters. 1, the posterior protocerebral commissure linking the POTU; 2, the median protocerebral commissure linking the CB and bilateral SIP; 3, the posterior great commissure linking the bilateral AVLP and the CL; 4, the LAL commissure; 5, the dorsal protocerebral commissure linking the bilateral AL and the SLP; 6, the anterior protocerebral commissure linking the bilateral tritocerebrum. Scale bar, 100 μm.

The brain of *H. armigera* larvae contains three distinct neuromeres, i.e., tritocerebrum (TR), deutocerebrum (DE), and protocerebrum (PR; Tang et al., 2014). The tritocerebrum is located most ventrally in the brain, and the deutocerebrum is located above the tritocerebrum (Figures 2, 3). The deutocerebrum consists of the antennal lobe (AL) and the antennal mechanosensory and motor center (AMMC; Figures 2, 3). The protocerebrum is the largest part of the brain and is located dorsally. Within the protocerebrum, the neuropils of the optic lobe (OL), mushroom body (MB), central body (CB), protocerebral bridge (PB), and lateral accessory lobe (LAL) were most prominent and easiest to identify (Figures 2, 3). In larvae the OL is located most lateral of the protocerebrum, the CB is in the center, and the calyx (CA) of MB is located most dorsally (Figures 2A–C). By referring to the detailed three-dimensional reconstructed brain maps of the fruit fly *Drosophila melanogaster* (Ito et al., 2014), a large number of homologous neuropils in the remaining part of the central brain were also identified. The superior neuropils contained the superior medial protocerebrum (SMP), the superior intermediate protocerebrum (SIP), the superior lateral protocerebrum (SLP), and the LH (Figures 2, 3B,C). The lateral neuropils comprise the inferior lateral protocerebrum (ILP), the anterior ventrolateral protocerebrum (AVLP), the posterior ventrolateral protocerebrum (PVLP), the posterolateral protocerebrum (PLP), and the posterior optic tubercle (POTU; Figures 2, 3C,E). The inferior neuropils include the crepine (CRE), clamp (CL), and inferior bridge (IB; x). The ventromedial neuropils include the posterior slope (PS) and the gorget (GOR; Figures 2, 3F,H).

### Number of Serotonin-Immunoreactive Cell Bodies in the *H. armigera* Larval Brain

**Figure 4:**
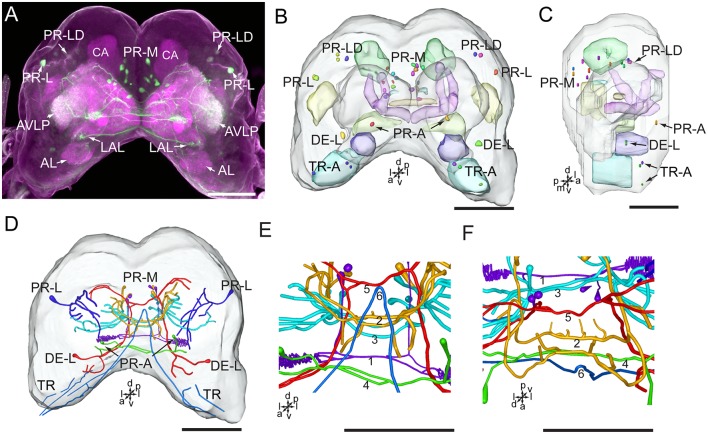
Confocal image and three-dimensional reconstructions showing the distribution of the serotonin-immunoreactive neuronal processes and cell bodies in the brain. **(A)** Projection view of the confocal stack images. **(B)** Three-dimensional reconstructions of the brain including the labeled cell body clusters in frontal view. **(C)** Three-dimensional reconstructions of the brain including labeled cell body clusters in sagittal view. **(D)** Reconstructed skeleton trees of the thick neuronal processes showing their projection patterns in frontal view. **(E)** Reconstructed skeleton trees of 1–6 commissures in frontal view. **(F)** Reconstructed skeleton trees of 1–6 commissures in dorsal view. AL, antennal lobe; AVLP, anterior ventrolateral protocerebrum; CA, calyx; LAL, lateral accessory lobe; TR, tritocerebrum. DE-L, PR-A, PR-L, PR-LD, PR-M, and TR-A are cell clusters. 1, the posterior protocerebral commissure linking the POTU; 2, the median protocerebral commissure linking the CB and bilateral SIP; 3, the posterior great commissure linking the bilateral AVLP and the CL; 4, the LAL commissure; 5, the dorsal protocerebral commissure linking the bilateral AL and the SLP; 6, the anterior protocerebral commissure linking the bilateral tritocerebrum. Directions: a, anterior; d, dorsal; l, lateral; p, posterior; v, ventral. Scale bars, 100 μm.

Serotonin-immunoreactivity was detected in a large area of the brain in *H. armigera* larvae (Figures 3, 4). All detected cell bodies were labeled and counted, and thick fibers were traced (Figure 4; Table 1). There were about 30–38 serotonin-immunoreactive cell bodies distributed singly or in clusters in both hemispheres of the brain (Figures 4A–C; Table 1). The cluster of PR-M contained the largest number of serotonin-immunoreactive cell bodies, about 10 in each hemisphere, distributed in the medial region of the posterior protocerebrum, ventrolateral to the calyx (Figures 4B,C; Table 1). The cluster of PR-LD, contained about three cell bodies in each hemisphere, located dorsally to the lateral protocerebral neuropil (Figures 4B,C; Table 1). The serotonin staining in these cell bodies was weak. The cluster of PR-L contained one cell body in each hemisphere, located laterally to the lateral protocerebral neuropil (Figure 4B; Table 1). Among seven preparations counted, however, there was one individual preparation containing two cell bodies in one cluster of PR-L. The cluster of PR-A is located anteriorly to the protocerebrum and dorsally to the AL (Figure 4B). We observed a single brightly stained serotonin-immunoreactive cell body in each hemisphere in the cluster of PR-A (Figures 4B,C; Table 1). In some preparations, however, we observed an additional weakly stained cell in the cluster of PR-A (Table 1).

**Table 1:** Number, location, and innervation area of serotonin-immunoreactive neurons in the brain and the gnathal ganglion of *Helicoverpa armigera* larvae.

	Cell body cluster	Number of neurons (*n*)	Location of cell body	Innervation areas
Brain	PR-M	17–20 (7)	Medial region of posterior protocerebrum, ventrolateral to the calyx	CB, bilateral AVLP, CL, CRE, SIP, POTU, and LAL
	PR-LD	5–6 (7)	Dorsolateral protocerebrum	Not resolved
	PR-L	2–3 (7)	Lateral protocerebrum, dorsolateral to OL	Ipsilateral OL, PLP, PVLP, SLP, and LAL
	PR-A	2–4 (7)	Anterior to protocerebrum, dorsal to AL	Bilateral LAL
	DE-L	2 (7)	Lateral to AL	Contralateral AL and bilateral SLP
	TR-A	6 (2)	Anteromedial tritocerebrum	Not resolved
GNG	GNG-AD	3–4 (7)	Anterior dorsal gnathal ganglion	Anterior mandibular neuromere and tritocerebrum
	GNG-AV	2 (7)	Anterior ventral gnathal ganglion	Not resolved
	GNG-M	3–4 (6)	Medial dorsal gnathal ganglion	Not resolved
	GNG-L1	4 (7)	Anterior lateral gnathal ganglion	Mandibular neuromere and tritocerebrum
	GNG-L2	4 (7)	Medial lateral gnathal ganglion	Maxillary neuromere and tritocerebrum
	GNG-L3	4 (7)	Posterior lateral gnathal ganglion	Labial neuromere and tritocerebrum

*n, the number of preparations used to count the number of cell bodies. AL, antennal lobe; AVLP, anterior ventrolateral protocerebrum; CB, central body; CL, clamp; CRE, crepine; ILP, inferior lateral protocerebrum; LAL, lateral accessory lobe; OL, optic lobe; PLP, posterior lateral protocerebrum; POTU, posterior optic tubercle; PVLP, posterior ventrolateral protocerebrum; SIP, superior intermediate protocerebrum; SLP, superior lateral protocerebrum*.

The cluster of DE-L, located laterally to the AL, contained a single serotonin-immunoreactive cell body in each hemisphere (Figure 4B; Table 1). In the tritocerebrum, we observed three weakly stained serotonin-immunoreactive cell bodies in each hemisphere in the cluster of TR-A, located anteriorly to the tritocerebrum (Figure 4B; Table 1). In some preparations, we did not detect serotonin-immunoreactive the cells in the cluster of TR-A.

### Innervation Patterns of Serotonin-Immunoreactive Neurons in the Brain

The low number of serotonin-immunoreactive neurons gives rise to many neuronal processes, spreading widely in the brain. Except the neurons in the cluster of PR-L, the identified neurons projected across the two brain hemispheres *via* commissures and innervated the contralateral brain hemisphere (Figures 4A,D). The bilateral serotonin-immunoreactive neurons form at least six commissures: (1) the posterior protocerebral commissure has processes to the POTU; (2) the median protocerebral commissure has processes to the SIP and the CB; (3) the posterior great commissure has processes to the AVLP and the clamp; (4) the LAL commissure has processes to the LAL; (5) the dorsal protocerebral commissure has processes to the SLP and the AL; and (6) the anterior protocerebral commissure connects the tritocerebra of two brain hemispheres (Figures 3, 4E,F).

**Figure 5:**
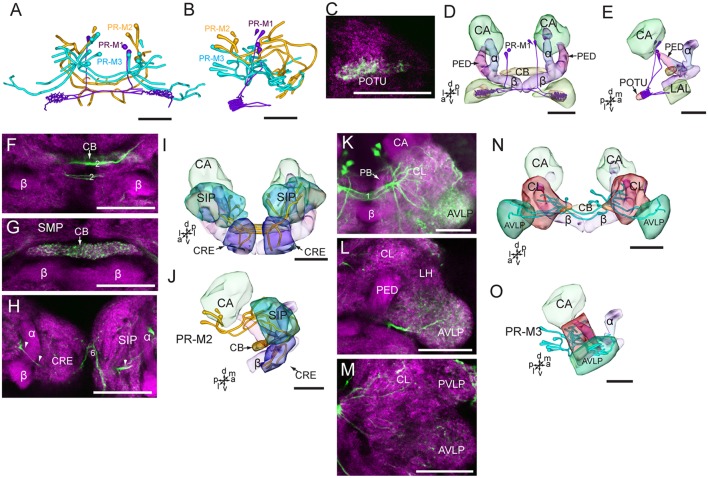
Confocal images and three-dimensional reconstructions showing the distribution of the serotonin-immunoreactive neuronal processes and cell bodies in the protocerebrum. **(A)** Reconstructed skeleton trees of PR-M neurons in frontal view. **(B)** Reconstructed skeleton trees of PR-M neurons in dorsal view. **(C)** Confocal image showing the serotonin-immunoreactive processes (green) in the POTU. **(D)** Three-dimensional reconstructions of serotonin-immunoreactive neurons in the cluster of PR-M1 and related neuropils in frontal view. **(E)** Three-dimensional reconstructions of the serotonin-immunoreactive neurons of PR-M1 and related neuropils in lateral view. **(F–H)** Confocal images showing the serotonin-immunoreactive processes of neurons PR-M2 (green). **(I)** Three-dimensional reconstructions of the serotonin-immunoreactive neurons in the cluster of PR-M2 and related neuropils in frontal view. **(J)** Three-dimensional reconstructions of the serotonin-immunoreactive neurons in the cluster of PR-M2 and related neuropils in lateral view. **(K–M)** Confocal images showing the serotonin-immunoreactive processes of neurons PR-M3 (green). **(N)** Three-dimensional reconstructions of the serotonin-immunoreactive neurons in the cluster of PR-M3 and related neuropils in frontal view. **(O)** Three-dimensional reconstructions of the serotonin-immunoreactive neurons in the cluster of PR-M3 and related neuropils in lateral view. α, alpha lobe; AVLP, anterior ventrolateral protocerebrum; β, belta lobe; CA, calyx; CB, central body; CL, clamp; CRE, crepine; LAL, lateral accessory lobe; PB, protocerebral bridge; PED, pedunculus; POTU, posterior optic tubercle; PVLP, posterior ventrolateral protocereburm; SIP, superior intermediate protocerebrum; SMP, superior medial protocerebrum. PR-M1-3 are cell clusters. 2, the median protocerebral commissure linking the CB and bilateral SIP; 6, the anterior protocerebral commissure linking the bilateral tritocerebrum. Directions: a, anterior; d, dorsal; l, lateral; m, medial; p, posterior; v, ventral. Scale bars, 50 μm.

In accordance with the innervation terminal regions, the cells in the cluster of PR-M were classified as PR-M1, PR-M2, and PR-M3. The cells of PR-M1, about four, located in the anterior part of the cluster of PR-M, have bilaterally symmetrical processes throughout the POTU (Figures 5A–E). A side branch projects further anteriorly from the POTU to the LAL (Figure 5E). The cells of PR-M2, about four, located in the medial part of the cluster of PR-M, have symmetrical processes in the SIP, the crepine, and the CB (Figures 5F–J). Two thick neurites originating in these cells run parallelly and form the medial protocerebral commissure nearby the CB (Figure 5F). One neurite gives rise to fine processes in CB, and is uniform throughout (Figure 5G). One neurite runs bypass the α lobe and gives rise to arborizations in the crepine (Figure 5H). The cells of PR-M3, about 12, located in the posterior part of the cluster of PR-M, have bilaterally symmetrical processes throughout the AVLP and the clamp (Figures 5K–O). The thick neurites originating from these cells run parallelly and form the posterior great commissure (Figure 5A). It was difficult to identify a single neuron to trace the processes and examine the innervation pattern in the ipsilateral and contralateral brain regions.

**Figure 6:**
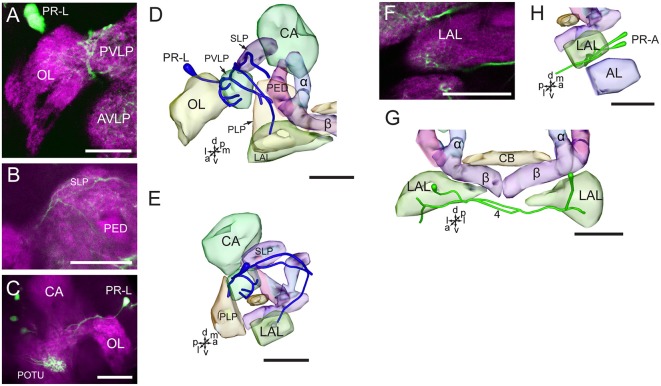
Confocal images and three-dimensional reconstructions showing the distribution of the serotonin-immunoreactive neuronal processes and cell bodies in the cluster of PR-L and PR-A in the protocerebrum. **(A–C)** Confocal images showing the serotonin-immunoreactive processes of neurons PR-L (green). **(D)** Three-dimensional reconstructions of the serotonin-immunoreactive neurons in the cluster of PR-L and related neuropils in frontal view. **(E)** Three-dimensional reconstructions of the serotonin-immunoreactive neurons in the cluster of PR-L and related neuropils in lateral view. **(F)** Confocal image showing the serotonin-immunoreactive processes of neurons PR-A (green). **(G)** Three-dimensional reconstructions of the serotonin-immunoreactive neurons in the cluster of PR-A and related neuropils in frontal view. **(H)** Three-dimensional reconstructions of the serotonin-immunoreactive neurons in the cluster of PR-A and related neuropils in lateral view. α, alpha lobe; AL, antennal lobe; AVLP, anterior ventrolateral protocerebrum; β, belta lobe; CA, calyx; CB, central body; LAL, lateral accessory lobe; PED, pedunculus; PLP, posterior lateral protocerebrum; POTU, posterior optic tubercle; PVLP, posterior ventrolateral protocereburm; SLP, superior lateral protocerebrum. PR-A and PR-L are cell clusters. 4, the LAL commissure. Directions: a, anterior; d, dorsal; l, lateral; m, medial; p, posterior; v, ventral. Scale bars, 50 μm.

The neurites of cells in the cluster of PR-L run medially and give rise to several branches projecting to different regions in the ipsilateral brain hemisphere, including the OL, the LAL, the SLP, the PVLP, and the posterior lateral protocerebrum (Figures 6A–E). Neurites from the cells in the cluster of PR-A form the LAL commissure and project to the anterior and ventral portions of the LAL (Figures 6F–H).

**Figure 7:**
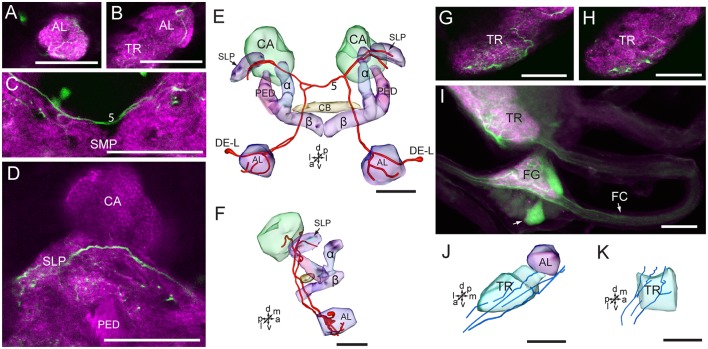
Confocal images and three-dimensional reconstructions showing the distribution of serotonin-immunoreactive neuronal processes in the protocerebrum, deutocerebrum and tritocerebrum. **(A–D)** Confocal images showing the serotonin-immunoreactive processes of neurons DE-L (green). **(E)** Three-dimensional reconstructions of the serotonin-immunoreactive neurons in the cluster of DE-L and related neuropils in frontal view. **(F)** Three-dimensional reconstructions of the serotonin-immunoreactive neurons in the cluster of DE-L and related neuropils in lateral view. **(G,H)** Confocal images showing the serotonin-immunoreactive processes in the tritocerebrum (green). **(I)** Confocal image showing the serotonin-immunoreactive processes in the tritocerebrum and the frontral ganglion (green). **(J)** Three-dimensional reconstructions of the serotonin-immunoreactive processes in the tritocerebrum and related neuropils in frontal view. **(K)** Three-dimensional reconstructions of serotonin-immunoreactive processes in the triotocerebrum in lateral view. α, alpha lobe; AL, antennal lobe; β, belta lobe; CA, calyx; FC, frontal connective; FG. Frontal ganglion; PED, pedunculus; SLP, superior lateral protocerebrum; SMP, superior medial protocerebrum; TR, tritocerebrum. DE-L are cell bodies. 5, the dorsal protocerebral commissure linking the bilateral AL and the SLP. Directions: a, anterior; d, dorsal; l, lateral; m, medial; p, posterior; v, ventral. Scale bars, 50 μm.

The two cells in the cluster of DE-L are deutocerebral neurons, they project dorsoposteriorly into the ipsilateral protocerebrum *via* the medial AL tract, crossed the midline dorsal to the CB, and extend to the contralateral AL, where innervated the entire AL (Figures 7A–F). The cells also extended some fine branches in the bilateral SLP, ventral to the calyx of the MB (Figure 7D).

**Figure 8:**
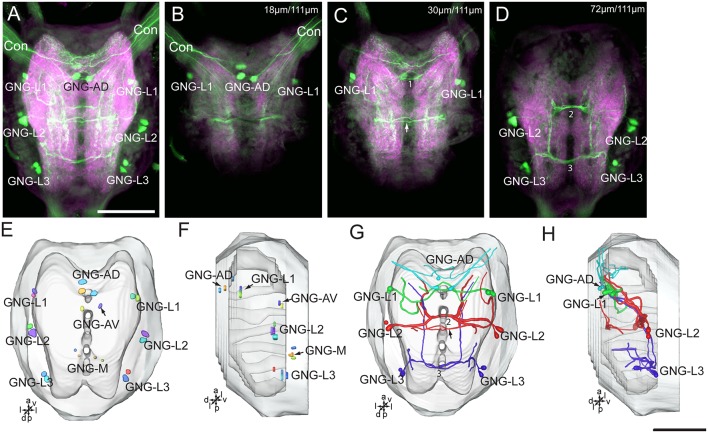
Confocal images and three-dimensional reconstructions showing the distribution of the serotonin-immunoreactive neuronal processes and cell bodies in the gnathal ganglion. **(A)** Projection view of confocal stack images of the gnathal ganglion. Serotonin-immunoreactive neurons in green and the surrounding neuropil in magenta. **(B–D)** Serial confocal images of the gnathal ganglion at different depths. **(E)** Three-dimensional reconstructions of the gnathal ganglion including labeled cell body clusters in frontal view. Arrow in **(C)** indicated the dorsal commissure formed by neurons of GNG-L2. **(F)** Three-dimensional reconstructions of the gnathal ganglion including the labeled cell body clusters in lateral view. **(G)** Reconstructed skeleton trees of the thick neuronal processes showing their projection patterns in frontal view. Arrow indicates the dorsal commissure formed by neurons of GNG-L2. **(H)** Reconstructed skeleton trees of the thick neuronal processes showing their projection patterns in lateral view. Con, connective; GNG-L1–L3, GNG-AD, GNG-AV, and GNG-M are cell clusters. 1, the commissure formed by neurons of GNG1; 2, the commissure formed by neurons of GNG-L2; 3, the commissure formed by neurons of GNG-L3. Directions: a, anterior; d, dorsal; l, lateral; p, posterior; v, ventral. Scale bars, 100 μm.

The cells in the cluster of PR-LD, and TR-A were weakly stained, and we were not able to detect neuronal processes from these cells. In the tritocerebrum, we observed some serotonin-immunoreactive neuronal processes, which may originate in the frontal ganglion and the gnathal ganglion (Figures 7G–K, 8). In the frontal ganglion, there is a single big serotonin-immunoreactive neuron, and some neurites connect to the tritocerebrum through the frontal connectives (Figure 7I).

The wide-field serotonin-immunoreactive neurons spread fine processes throughout the brain, however, some regions of neuropils lacked serotonin, including the calyx, the pedunculus, α lobe, β lobe, the PB, the lateral horn, and the medial and dorsal portions of the LAL (Figures 5F,G,K,L, 6B,C,F, 7D).

### Serotonin-Immunoreactivity in the Gnathal Ganglion of *H. armigera* larvae

There are about 20–22 serotonin-immunoreactive neurons in the gnathal ganglion, distributed in several clusters; each cluster contains 2–4 neurons (Table 1; Figure 8). The cluster of GNG-AD contains 3–4 cell bodies, located in the medial region of the anteriodorsal gnathal ganglion. The cluster of GNG-AV contains two neurons and GNG-M contains 3–4 neurons, located in the anterior and medial regions of ventral gnathal ganglion. The cells in these clusters were weakly stained, and no neurites extending from their cell bodies were detected. In each lateral side of the gnathal ganglion, there are three clusters, GNG-L1–GNG-L3; each cluster contains two neurons. The cells in the cluster of GNG-L1 are situated in the dorsal lateral gnathal ganglion, whereas GNG-L2 and GNG-L3 cells are located ventrolaterally.

Three cell clusters in the lateral gnathal ganglion, GNG-L1–GNG-L3, were associated with three neuromeres, respectively, i.e., mandibular neuromere, maxillary neuromere, and labial neuromere (Figures 8A–D,G,H). From two cells of one cluster on each side of the ganglion, major processes extend into the contralateral hemisphere *via* a commissure, which contained four neurites. In the mandibular neuromere, at least two branches extended from the cell; one laterally and the other medially. Both branches projected ventroanteriorly to the tritocerebrum through circumoesophageal connectives and had many fine arborizations in the medial regions of each gnathal neuromere hemisphere and tritocerebrum (Figures 7G,H, 8A–D,G,H). In the maxillary and labial neuromeres, the serotonin-immunoreactive neuron processes show a branching pattern similar to that of the mandibular neuromere. The branches run across the midline *via* the commissure and project through the proceeding neuromere, and terminate in the tritocerebrum. In addition to the prominent commissures in the ventral part of the maxillary and labial neuromeres, a dorsal immunoreactive commissure was observed at the dorsal surface of each neuromere (indicated by arrows in Figures 8C,G).

From each cell in the cluster of GNG-AD, at least one neurite projects to the tritocerebrum, exhibiting many fine immunoreactive arborizations in the anterior part of mandibular neuromere (Figures 8A,B,G,H). We were able not to detect serotonin-immunoreactive processes originating in the weakly stained cells in the cluster of GNG-AV and GNG-M (Table 1).

## Discussion

### Number of Serotonin-Immunoreactive Neurons in the Brain

For the first time, the serotonergic neurons in the brain and the gnathal ganglion of *H. armigera* larvae were comprehensively revealed by performing immunohistochemistry with anti-serotonin serum. There are about 40 serotonin-immunoreactive neurons in the brain and about 20 in the gnathal ganglion of *H. armigera* larvae. The numbers are similar to those reported in larvae of the sphinx moth, *Manduca sexta*, flies *D. melanogaster*, *Calliphora erythrocephala* and *Sarcophaga bullata*, and the beetle *Tenebrio molitor* (Table 2, Nässel and Cantera, 1985; Vallés and White, 1988; Granger et al., 1989; Griss, 1989; Breidbach, 1990; Huser et al., 2012). Compared to the large number of neurons in the brain and the gnathal ganglion, the number of serotonin-immunoreactive neurons is low. In adults of *M. sexta* and *D. melanogaster*, the number of serotonin-immunoreactive cell bodies in the gnathal ganglion is also about 20 (Table 2, Vallés and White, 1988; Homberg and Hildebrand, 1989a; Sitaraman et al., 2008), suggesting that the serotonin-immunoreactive neurons in larvae persist during metamorphosis to adults. Similarly, in the central brain of adults of these species, e.g., brain neuropils excluding the OL, the number of serotonin-immunoreactive cell bodies is about 40 (Vallés and White, 1988; Homberg and Hildebrand, 1989a; Sitaraman et al., 2008). Similar number were found in the honeybee *Apis mellifera*, the wasp *Trichogramma evanescens*, and the blood-feeding bug *Rhodnius prolixus*, which are lower than the number of 200 in the cockroach *Periplaneta americana* (Table 2, Klemm et al., 1984; Schürmann and Klemm, 1984; Lange et al., 1988; van der Woude and Smid, 2017). The OL in larvae (the larval optic center) is an OL anlage that develops during metamorphosis to the adult form with the neuropils of lamina, medulla, and lobula complex (Nässel et al., 1987; Breidbach, 1990; Seidel and Bicker, 1996; Tang et al., 2014). The differentiation of serotonin-immunoreactive neurons in the OL is dependent on the development of OL neuropils (Nässel et al., 1987). Most serotonin-immunoreactive cell bodies in the OL are local amacrine neurons that innervate the local neuropils of the OL (Homberg and Hildebrand, 1989b). There are about 600 serotonin-immunoreactive cell bodies located in the OL of *M. sexta* and 20 in *D. melanogaster* (Homberg and Hildebrand, 1989b; Sitaraman et al., 2008). For comparison, it is essential to examine the serotonin-immunoreactive neurons in adult *H. armigera* further. The similarity and difference in serotonin-immunoreactive neurons between the larvae and the adults could provide insight on the development and function of serotonin in different stages.

**Table 2:** Number of serotonin-immunoreactive neurons in the brain of different insect species.

	Species	Central brain	Gnathal ganglion	Reference
**Larva**				
Beetle	*Tenebrio molitor*	42	–	Breidbach (1990)
Moth	*Manduca sexta*	36–40	20	Griss (1989) and Granger et al. (1989)
Flies	*Drosophila melanogaster*	40	16	Vallés and White (1988)
	*Calliphora erythrocephala*	30	22	Nässel and Cantera (1985)
	*Sarcophaga bullata*	32	22	Nässel and Cantera (1985)
**Adult**				
Cockroach	*Periplaneta americana*	200	–	Klemm et al. (1984)
Bug	*Rhodnius prolixus*	40	13	Lange et al. (1988)
Beetle	*Tenebrio molitor*	42	–	Breidbach (1990)
Wasp	*Trichogramma evanescens*	36	22	van der Woude and Smid (2017)
Bee	*Apis mellifera*	50	–	Schürmann and Klemm (1984)
Moth	*Manduca sexta*	40	20	Homberg and Hildebrand (1989a)
Fly	*Drosophila melanogaster*	38–40	16–18	Vallés and White (1988), Sitaraman et al. (2008) and Huser et al. (2012)

*“-”, Not counted*.

### Serotonin-Immunoreactive Neurons Associated With the Protocerebrum

The location of serotonin-immunoreactive cell bodies and their branching patterns of *H. armigera* larvae are also similar to that of other studied insects (Vallés and White, 1988; Granger et al., 1989; Griss, 1989; Breidbach, 1990). Most serotonin-immunoreactive neurons are wide-field neurons and have processes extending throughout the neuropils in the brain. Although the morphology of these neurons is very similar, their terminals in part invade some different areas among different species, suggesting species-specific modifications of arborization patterns.

In the protocerebrum, the cells in the cluster of PR-M have processes projecting to the CB, and bilateral crepine, clamp, SIP, AVLP, and POTU. The arborizations in the CB, the clamp, the AVLP, and the POTU are quite dense. The cells in the cluster of PR-L have processes projecting mainly to the ipsilateral posterior ventrolateral, posterior lateral, and SLP. A few processes of these cells reach the inner part of the ipsilateral OL. The cells in the cluster of PR-A have processes and project bilaterally to the LAL. The pattern of serotonin-immunoreactive arborizations in the superior and lateral protocerebrum is roughly similar between species. Comparisons of these neuropils between different species are difficult to make due to the scarcity of information. Therefore, comparisons of the serotonin-immuonstaining between *H. armigera* and other species are mainly focused on the prominent neuropils, the MB, CB, the PB, the LAL, and the POTU. The strong serotonin-immunoreactivity in the POTU in larvae, however, was first found in *H. armigera*. Previously, the serotonin-immunoreactive processes in the POTU were found in adults of *M. sexta* and the locust *Schistocerca gregaria*, but the staining intensity was lower (Homberg and Hildebrand, 1989a; Homberg, 1991; Beetz et al., 2015). Serotonin-immunoreactive neurons linking the LAL are similar in several insect species, i.e., *D. meloanogaster*, *M. sexta*, and *T. molitor*, dung beetles *Scarabaeus lamarchki*, *S. satyrus*, *S. gragaria*, and the aphid *Acyrthosiphon pisum* (Vallés and White, 1988; Granger et al., 1989; Homberg and Hildebrand, 1989a; Breidbach, 1990; Homberg, 1991; Kollmann et al., 2011; Immonen et al., 2017). The presence of serotonin-immunoreactive processes in the CB also seems to be common across insect species. However, in *S. gregaria* and *P. americana*, the lower part of the CB is devoid of serotonin-immunoreactive processes (Klemm et al., 1984; Tyrer et al., 1984). The PB, which is associated with the CB, as a part of the central complex, showed no serotonin-immunoreactivity in *H. armigera* larvae. The PB of *M. sexta* larvae and adults, as well as the wasp *T. evanescens*, the ant *Harpegnathos saltator*, and *A. mellifera*, also lacks serotonin-immunoreactive processes (Schürmann and Klemm, 1984; Granger et al., 1989; Homberg and Hildebrand, 1989a; Hoyer et al., 2005; van der Woude and Smid, 2017). In contrast, the PB of *S. gregaria* and *P. americana* contains dense serotonin-immunoreactive processes (Klemm et al., 1984; Tyrer et al., 1984). The prominent neuropils of the MB, including the calyx, the pedunculus, and the α and β lobes, lack serotonin-immunoreactivity in *H. armigera* larvae. Similar findings have been reported in larvae of *M. sexta* and* D. melanogaster* (Granger et al., 1989; Huser et al., 2012). In contrast, the MB of adult *M. sexta* and *D. melanogaster* contains fine serotonin-immunoreactive processes (Homberg and Hildebrand, 1989a; Sitaraman et al., 2008). These results suggest that some immunoreactive neurons are remodeled during the metamorphosis from larva to adult in moths. In general, the intrinsic neurons of the MB, the Kenyon Cells, are devoid of serotonin across insect taxa, whereas extrinsic neurons of the MB show the considerably varied innervation patterns. For instance, the calyx of *A. mellifera*, the calyx collar of *H. saltator*, the calyx and the pedunculus of *S. gregaria* and *Locusta migratoria*, the inner part of the calyx and the upper part of the pedunculus of *P. americana*, and the pedunculus and the α and β lobes of *T. infestans* contain no serotonin-immunoreactive processes (Klemm et al., 1984; Schürmann and Klemm, 1984; Tyrer et al., 1984; Ignell, 2001; Settembrini and Villar, 2004; Hoyer et al., 2005). In addition, in the larva of *H. armigera*, the lateral horn, an area associated with the MB, lacks serotonin-immunoreactivity. To date, such findings have not been reported in other species.

### Serotonin-Immunoreactive Neurons Associated With Deutocerebrum

A pair of deutocerebral serotonin-immunoreactive DE-L neurons were first reported in larva of *M. sexta* (Kent et al., 1987). These deutocerebral neurons have arborizations in the contralateral AL and bilateral SLP. The AL in the larval brain is also called the larval antennal center. In adults, the branching pattern persists and expands in the AL with the formation of glomeruli (Kent et al., 1987). Ultrastructural studies on the synaptic terminals indicated that the deutocerebral neuron is a feedback neuron to the AL for olfactory processing (Sun et al., 1993). Electrophysiological recordings demonstrated that the deutocerebral serotonin-immunoreactive neuron showing responses to odorants and mechanical stimuli (Hill et al., 2002; Zhao and Berg, 2009). The serotonin-immunoreactive deutocerebral neurons were found in all studied species but varied in the number of cells and innervation patterns. There is a single cell body in each AL in the species of Lepidoptera, Trichoptera, Diptera, Coleoptera, and Neuroptera and 2–8 cells in each AL in Orthoptera and Blattaria (Dacks et al., 2006). In Hymenoptera, however, the immunoreactive cell body in AL is absent, and the processes in the AL originate in an ascending neuron. In Orthoptera and Blattaria, the processes of the deutocerebral neurons innervate the ipsilateral hemisphere of the AL and the protocerebrum (Dacks et al., 2006).

### Serotonin-Immunoreactive Neurons Associated With Tritocerebrum

The tritocerebrum is a largely reduced neuropil, which is hard to discriminate from the surrounding neuropils in the brain of many holometabolous insect species (Ito et al., 2014). In the larvae of *H. armigera*, however, the tritocerebrum is a distinct and large neuropil, which is similar to that of hemimetabolous species, such as the locust *S. gregaria* and the bug *Apolygus lucorum* (Kurylas et al., 2008; Tang et al., 2014; Xie et al., 2016). The cells in the cluster of TR-A in tritocerebrum were weakly stained with anti-serotonin serum and their neuronal processes were not detected. Throughout the tritocerebrum, however, serotonin-immunoreactive neuronal processes are abundant, and they may have multiple origins, including the frontal ganglion and the gnathal ganglion. A commissure in the front of the medial protocerebrarum formed by neurons which links the tritocerebrum, giving rise to some arborizations in the vicinity. Although the number of studies in other species is low, the serotonin-immunoreactivity in the tritocerebrum seems to be common across insect taxa (Nässel, 1988; Granger et al., 1989; Wegerhoff, 1999). The serotonin-immunoreactive neuronal processes in the tritocerebrum connect the protocerebrum, deutocerebrum, gnathal ganglion, and stomatogastric nervous system.

### Serotonin-Immunoreactive Neurons Associated With Gnathal Ganglion

In the gnathal ganglion, the serotonin-immunoreactive neurons also show high conservation across insect taxa. All three neuromeres of the gnathal ganglion in *H. armigera* larvae, i.e., the mandibular, maxillary, and labial neuromeres contain widespread processes originating in serotonin-immunoreactive cell clusters on both sides. The thick processes form a horseshoe pattern, cross the midline *via* a commissure and project anteriorly to the contralateral tritocerebrum. Such neurons and their branching patterns were also found in larvae of *M. sexta*, *T. molitor* and the flies, *D. melanogaster*, *C. erythrocephala*, *S. bullata* (Nässel and Cantera, 1985; Griss, 1989; Breidbach, 1990; Huser et al., 2012). In adults of these species, serotonin-immunoreactive neurons and processes in the gnathal ganglion well persist during metamorphosis. Similar neurons were also found in *S. gregaria*, *A. mellifera*, and *P. americana* (Bishop and O’Shea, 1983; Tyrer et al., 1984; Rehder et al., 1987; Vallés and White, 1988; Griss, 1989; Homberg and Hildebrand, 1989a; Breidbach, 1990). Each cell cluster contains two cell bodies. In flies, however, there are 2–5 cell bodies in each cluster (Nässel and Cantera, 1985; Vallés and White, 1988; Huser et al., 2012). In addition, there are 3–4 large serotonin-immunoreactive cells the medial gnathal ganglion. Similar results were also found in *S. gregaria*, *P. americana*, and larval and adult* M. sexta* (Bishop and O’Shea, 1983; Tyrer et al., 1984; Griss, 1989; Homberg and Hildebrand, 1989a). They were identified as efferent neurons in the mandibular neuromere (Tyrer et al., 1984; Griss, 1989; Homberg and Hildebrand, 1989a). Intracellular recordings from such neurons of *M. sexta* larvae revealed overshooting soma spikes of large amplitude and long duration, which suggest these neurons are neurosecretory cells (Griss, 1989). In addition, in the gnathal ganglion of *S. gregaria*, four serotonin-immunoreactive neurons innervate the salivary gland (Tyrer et al., 1984); these neurons have not been found in *H. armigera* larvae.

In *H. armigera* larvae, compared to the brain, the neuropil volume of the gnathal ganglion is smaller and the sub-regions are fewer (Tang et al., 2014). Correspondingly, the number of serotonin-immunoreactive neurons in the gnathal ganglion is lower and neuronal branch patterns are more concise.

## Conclusion

We have provided, for the first time, a comprehensive description of the serotonergic neuronal network in *H. armigera* larvae. In accordance with its widespread presence in insects, serotonin plays a variety of roles. In the present study, we found there are about 40 serotonin-immunoreactive neurons in the brain and about 20 in the gnathal ganglion. Most of these neurons are wide-field neurons giving rise to processes throughout the neuropils of the brain and the gnathal ganglion. In the central brain, serotonin-immunoreactive processes are present bilaterally in the tritocerebrum, the deutocerebrum, and major regions of the procerebrum, including the CB, the LAL, the clamp, the cripine, the superior protocerebrum, and the lateral protocerebrum. These results indicate that serotonin may play a variety of roles in *H. armigera*. Particularly, the AVLP, CB, and the POTU contain extensive serotonin-immunoreactive process terminals. The CB has been demonstrated to be a locomotion and navigation center, and the POTU is involved in navigation in locust (Pfeiffer and Homberg, 2014; Beetz et al., 2015). The AVLP in moths is a region involved in sound information processing (Pfuhl et al., 2014). The high serotonin-immunoreactivity in the CB, the POTU and the AVLP might indicate that serotonin plays important roles in *H. armigera* larvae for locomotion and sound reception. However, the MB, the lateral horn, and the PB are devoid of serotonin-immunoreactivity. The MB and the lateral horn are higher olfactory centers of insects. The absence of serotonin in these centers suggests that serotonin is not involved in the modulation of higher centers. Instead, serotonin has been demonstrated to modulate the olfactory information in the AL by giving the feedback to the protocerebrum (Kloppenburg and Mercer, 2008). In addition, the MB is the learning and memory center and serotonin has the function of learning and memory (Sitaraman et al., 2008, 2012). The absence of serotonin in the MB indicates serotonin may play no role in learning and memory in *H. armigera* larvae. In the gnathal ganglion, the serotonin-immunoreactive processes are also widespread, and most, if not all, of the neurons project to the tritocerebrum. The gnathal ganglion is the gustatory center, while the tritocerebrum is the stomatogastric center. The links between serotonin-immunoreactive neurons from these two centers suggest the serotonin plays important roles in feeding, from selection to intake digestion.

In summary, the results of the present study provide a comprehensive description of the serotonergic neuronal network in *H. armigera* larvae, and show a map of the neural architecture and the distribution of neural substances, allowing us to explore the neural mechanisms of behaviors, such as host selection, navigation, and feeding preference and plasticity, by using electrophysiological and pharmacological approaches on the target regions.

## Data Availability

All datasets generated for this study are included in the manuscript.

## Author Contributions

Q-BT, W-BC and X-CZ: study concept, design and final manuscript. Q-BT, W-WS, Y-JC, G-YX and W-BC: acquisition of data. W-WS, G-YX, W-BC and X-CZ: analysis and interpretation of data. W-BC and X-CZ: drafting of the manuscript. Q-BT and X-CZ: obtained funding.

## Conflict of Interest Statement

The authors declare that the research was conducted in the absence of any commercial or financial relationships that could be construed as a potential conflict of interest.
